# Atomistic Insights into the Hydration Structure of Stachyose: A Minimum-Distance Distribution Function Approach

**DOI:** 10.3390/molecules31213799

**Published:** 2026-10-22

**Authors:** Xiankai Jiang, Yuqiao Shen, Xiaolei Yin, Changying Wang, Junjian Miao

**Affiliations:** 1 School of Sciences, Changzhou Institute of Technology, Changzhou 213032, China; jiangxk@czu.cn (X.J.); shenyq@czu.cn (Y.S.); yinxl@czu.cn (X.Y.); wcy58462006@126.com (C.W.); 2 College of food Science and Technology, Shanghai Ocean University, Shanghai 201306, China; 3 Engineering Research Center of Food Thermal-Processing Technology, Shanghai Ocean University, Shanghai 201306, China

**Keywords:** stachyose, minimum-distance distribution function, molecular dynamics, water bridge, residence time, carbohydrate solvation

## Abstract

Stachyose, a raffinose-family tetrasaccharide abundant in legumes, exhibits significant prebiotic and anti-nutritional activities that are intimately linked to its aqueous solvation behavior. Yet, the atomistic details of how water molecules organize around this non-globular, flexible tetrasaccharide remain poorly understood, largely because traditional radial distribution functions fail for molecules lacking a well-defined geometric center. Here, we employ molecular dynamics simulations (3 independent 300 ns replicas, GLYCAM06j/TIP3P) in conjunction with the minimum-distance distribution function (MDDF) framework to dissect the hydration structure of stachyose at multiple length- and time-scales. Our results reveal a densely populated first hydration shell (3.5 Å) comprising 69.5 ± 0.3 water molecules, with a pronounced MDDF peak at 1.86 Å characteristic of O–H···O hydrogen bonding. Direct hydrogen-bond analysis identifies an average of 21.1 ± 0.1 solute–water H-bonds per frame, while residence-time calculations show that 11.1 ± 0.1 water molecules remain within the 5.5 Å shell for longer than 200 ps, forming a dynamic hydration core. Notably, DBSCAN cluster analysis of first-shell waters reveals a percolated hydrogen-bonded network: the largest instantaneous cluster spans 53–58 of the ~70 first-shell waters, yet the network is compositionally fluid, with individual waters exchanging on a ~40 ps timescale and only ~16% of shell waters being isolated at any instant. Conformational clustering further resolves four major families (89.2% of all frames), with terminal residues (2CU and 0LA) exhibiting higher solvent exposure than internal ones. Surprisingly, the fructose terminus (0LA) shows a dynamic hydration contribution (29.0%) essentially equal to that of the galactose terminus (28.6%) despite a smaller hydrophilic fraction (65.9% vs. 70.0% for the galactose terminus), underscoring the limitation of geometry-only descriptors. These findings provide a quantitative, residue-resolved picture of stachyose solvation and establish a computational workflow applicable to complex carbohydrates in general.

## 1. Introduction

Raffinose-family oligosaccharides (RFOs), including stachyose, raffinose, and verbascose, are abundant in soybeans, lentils, lupins, and other legumes [1,2]. They are recognized as important prebiotics that selectively promote the growth of beneficial gut microbiota [3,4,5], yet their presence in food matrices also contributes to flatulence and reduced mineral bioavailability because humans lack α-galactosidase in the small intestine [4,6]. The biological fate of RFOs—whether they are fermented in the colon or interact with digestive enzymes and mucosal surfaces—is governed not only by their covalent structure but also by their aqueous solvation architecture, which shapes the conformational ensemble, the exposure of glycosidic bonds to hydrolases, and the accessibility of hydroxyl groups to hydrogen-bonding partners [7,8].

More broadly, the hydration shell is the primary medium through which oligosaccharides interact with their biological environment. Carbohydrate–protein recognition, for instance, is frequently mediated or modulated by water: structural and thermodynamic studies of lectin–sugar complexes have shown that bound water molecules can account for a substantial fraction of the binding interface and, in some cases, confer specificity itself through bridging interactions [9,10]. The conformational ensemble that an oligosaccharide presents to its receptors is likewise solvent-conditioned, because rotation about glycosidic linkages reflects a subtle balance among intramolecular hydrogen bonds, exo-anomeric effects, and competition from bulk solvent [7,8,11]. Hydration also underlies the protective action of sugars toward labile biomolecules: the exceptional bioprotectant trehalose owes part of its efficacy to an anomalously high hydration number and to its capacity to perturb the hydrogen-bond network of surrounding water well beyond the first shell [12,13,14,15]. A quantitative, atomistically resolved description of how water organizes around a given sugar is therefore a prerequisite for understanding carbohydrate function in food science, gut physiology, and plant stress biology.

Experimentally, carbohydrate hydration has been probed by ^17^O NMR relaxation [16], combined NMR and molecular modeling studies [8,17], terahertz absorption spectroscopy [15,18], and volumetric or water-activity determinations of hydration numbers [19]. These approaches converge on the view that each dissolved sugar molecule dynamically perturbs tens to hundreds of water molecules, but they offer limited spatial resolution and cannot assign hydration features to individual residues or functional groups. Molecular dynamics (MD) simulations fill this gap: since the seminal simulations of Brady on glucose and maltose [20,21], MD has been used to characterize hydration numbers, hydrogen-bond statistics, stereochemical effects on hydration [22], and sugar-induced retardation of water dynamics [17,23] for mono- and disaccharides, supported by steadily refined carbohydrate force fields [24,25,26,27]. Extending such atomistic analyses to larger, flexible oligosaccharides such as stachyose, however, poses a specific methodological difficulty that concerns the very definition of the hydration shell.

The hydration structure of carbohydrates has traditionally been characterized through radial distribution functions (RDFs) or solvent-accessible surface area (SASA) calculations [28,29,30]. For non-globular, flexible molecules such as stachyose, the RDF suffers from a fundamental ambiguity: it requires a choice of geometric center (e.g., the center of mass), which does not coincide with any chemically meaningful “surface” [31]. Consequently, the first peak of a center-referenced RDF may reflect the average shape of the molecule rather than true solvent–solute contact distances. This limitation is particularly severe for linear or branched oligosaccharides, whose different residues project into the solvent to vastly different extents [7,23].

The minimum-distance distribution function (MDDF), implemented in the ComplexMixtures.jl package [31], circumvents this problem by computing, for each frame, the distribution of the minimum distance between any solute atom and any solvent atom. The resulting distribution is inherently shape-independent, connects directly to Kirkwood–Buff solvation thermodynamics [32], and can be decomposed into atomic- or residue-level contributions, offering a natural “solvent-shell” perspective [31,32]. In this study, we combine 900 ns of explicit-solvent MD simulations (three independent 300 ns replicas) with MDDF analysis, hydrogen-bond profiling, residence-time measurements, and conformational clustering to construct a multi-layered, atomistically resolved picture of stachyose hydration. Specifically, we address the following questions: (i) What is the thickness and population of the first hydration shell of stachyose? (ii) How do the four monosaccharide residues differ in their solvent exposure? (iii) What are the lifetime and connectivity pattern of hydration waters—do they form stable bridges or isolated contacts? (iv) How does molecular flexibility modulate the static geometric picture provided by SASA?

## 2. Materials and Computational Methods

### 2.1. System Preparation and Molecular Dynamics Simulations

The initial structure of stachyose (α-D-galactopyranosyl-(1→6)-α-D-galactopyranosyl-(1→6)-α-D-glucopyranosyl-(1→2)-β-D-fructofuranose, i.e., 0LA-6LA-6GA-2CU) was built and geometry-optimized using the GLYCAM06j force field [24] within the AmberTools26 suite. The molecule was solvated in a cubic TIP3P [33] water box with a minimum clearance of 15.0 Å from the solute to the box edge, yielding approximately 2579 water molecules. No counter-ions were added, as stachyose is electrically neutral. The system was subjected to energy minimization (5000 steps), followed by 1 ns NVT equilibration and NPT pre-equilibration until the density converged to 0.99–1.02 g cm^−3^. Production runs were performed in the NPT ensemble at 300 K and 1 bar using the Langevin thermostat (γ = 2.0 ps^−1^) and the Monte Carlo barostat, with a 2 fs time step, SHAKE constraints [34] on all bonds involving hydrogen, and a 10.0 Å real-space cutoff with particle-mesh Ewald electrostatics. Three independent production replicas of 300 ns each (900 ns in total) were launched from the pre-equilibrated NPT structure with freshly generated random initial velocities, and coordinates were saved every 10 ps in NetCDF format with imaging enabled. The first 1 ns of each replica was discarded as re-equilibration, leaving 29,900 frames per replica for analysis. Temperature (300 ± 3 K), density (0.989 ± 0.002 g cm^−3^), solute RMSD, and radius of gyration were monitored in every replica to confirm stability and independent sampling. Unless otherwise noted, all reported values are means ± standard deviation across the three independent replicas. All MD simulations were carried out using pmemd.cuda (Amber 26) [35].

Control simulations of sucrose. To validate the treatment of the α-D-Glcp-(1→2)-β-D-Fruf linkage, an isolated sucrose molecule was built with the GLYCAM06j force field, solvated in a cubic TIP3P water box (minimum solute–box clearance 15.0 Å), and simulated with a protocol identical to the stachyose production runs (300 K, 1 bar, Langevin thermostat with γ = 2.0 ps^−1^, Monte Carlo barostat, 2 fs time step, SHAKE constraints, 10.0 Å real-space cutoff with particle-mesh Ewald electrostatics). Three independent production replicas of 300 ns each (900 ns in total) were collected, and the first 1 ns of each replica was discarded. Glycosidic torsions are reported in the heavy-atom convention φ = O5g–C1g–O2f–C2f and ψ = C1g–O2f–C2f–O5f, identical to that of the reference GLYCAM06 sucrose study [36]; the glucopyranose ring conformation was monitored through the Cremer–Pople puckering coordinate θ, and the fructofuranose ring through the Altona–Sundaralingam pseudorotation phase angle. Results of the control simulations are reported in Section 3.5 and in Section S1 of the Supporting Information.

### 2.2. Minimum-Distance Distribution Function (MDDF) Analysis

The MDDF and associated Kirkwood–Buff (KB) integrals were computed using ComplexMixtures.jl (v2.18) [31]. The solute was defined as all non-water atoms belonging to a single stachyose molecule, and the solvent as TIP3P water molecules. The bulk-range distance was set to 8.0–12.0 Å for the primary analysis, with additional calculations at 12.0–16.0 Å and 15.0–20.0 Å to assess KB convergence [32]. The MDDF was decomposed into per-residue and per-atom-type contributions using the built-in group-decomposition feature, with solute atoms grouped into chemically meaningful selections based on atom indices. All trajectory processing was performed with Chemfiles.jl, which natively reads Amber NetCDF format.

### 2.3. Hydrogen-Bond and Geometric Hydration Analysis

Direct solute–water hydrogen bonds were identified using cpptraj (AmberTools26) [37] with a donor–acceptor distance cutoff of 3.0 Å and an angle cutoff of 135°. Two directional classes were distinguished: (i) water as H-bond donor to solute oxygen acceptors, and (ii) solute hydroxyl groups as donors to water oxygen acceptors. The geometric hydration number (coordination number) was obtained from a radial solvent-shell counting analysis at cutoffs of 3.5, 5.5, and 7.0 Å, taking into account all atoms of the complete stachyose molecule (all four residues).

### 2.4. Water Residence Time and Cluster Analysis

Water residence times within the 5.5 Å solvation shell were computed using a custom Python script based on MDAnalysis [38]. For each water molecule, the minimum distance to any solute atom was calculated with periodic boundary conditions. With coordinates saved every 10 ps, a water molecule was considered long-resident if it remained within the shell for at least 20 consecutive frames (200 ps); transient excursions outside the shell lasting no more than two frames (20 ps) were bridged and counted as continuous residence [39,40]. This gap tolerance prevents instantaneous boundary recrossings at the 10 ps saving resolution from artificially truncating residence segments. Water clusters within the shell were identified using DBSCAN density-based clustering [41] with a clustering distance cutoff of 3.0 Å and a minimum cluster size of 2 water molecules, applied to the oxygen positions of first-shell (3.5 Å) water molecules extracted from the trajectory after whole-molecule imaging across periodic boundaries. Restricting the clustering to the first shell is essential: including second-shell waters allows bulk-like molecules to percolate into large, physically meaningless clusters.

### 2.5. Solvent-Accessible Surface Area and Conformational Clustering

SASA was calculated using a molecular-surface-area algorithm in cpptraj [37] for the entire solute and for individual residues. The hydrophilic/hydrophobic decomposition was performed using FreeSASA [42] with the Shrake–Rupley algorithm [30] on a representative trajectory frame. Hydrophilic area was defined as the sum of O-atom and hydroxyl-hydrogen contributions; hydrophobic area comprised C atoms and C–H hydrogens. Conformational clustering of the solute heavy atoms was performed with the average-linkage algorithm based on RMSD, with frames sampled every 10th frame from the trajectory, applied to the concatenated 900 ns ensemble to ensure a consistent cluster definition; the resulting cluster assignments are reported per replica in Section 3.5.

## 3. Results and Discussion

### 3.1. Overall Hydration Structure: MDDF and Coordination Numbers

**Figure 1:**
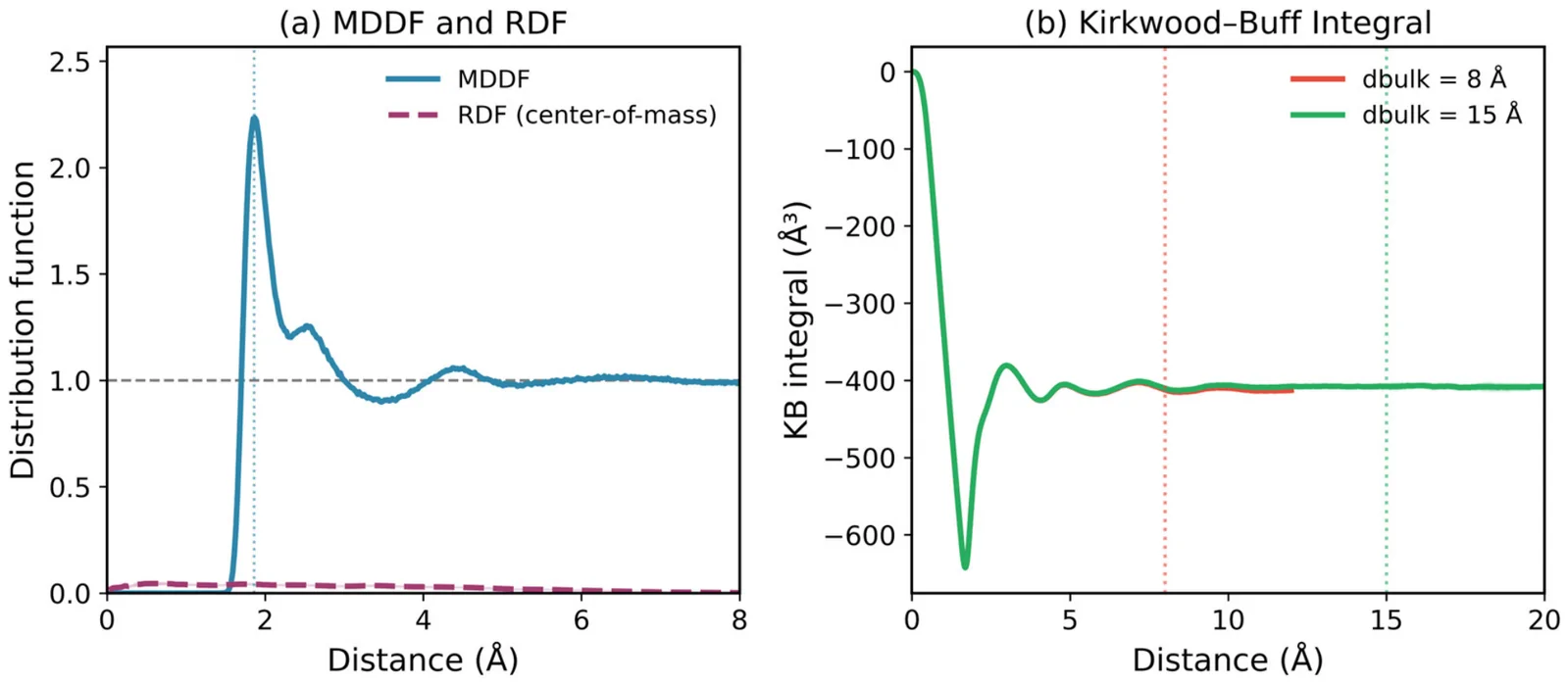
Hydration shell structure of stachyose. (**a**) Minimum-distance distribution function (MDDF, blue) and radial distribution function (RDF, red) of water around stachyose. The dashed gray line marks the bulk reference (MDDF = 1). The first peak at 1.86 Å corresponds to O–H···O hydrogen-bonding distances. (**b**) Kirkwood–Buff integral at two dbulk choices (8 Å, red; 15 Å, green), illustrating the excluded-volume effect at long range. The vertical dotted lines mark the corresponding dbulk positions, beyond which the solvent is assumed to behave as bulk (g(r) = 1).

The MDDF of water around stachyose exhibits a sharp first peak at 1.86 Å with a magnitude of 2.24 (Figure 1a), indicating that the first hydration layer is approximately 2.3 times denser than bulk water at contact distance. Because the MDDF records the minimum solute–solvent distance rather than a center-referenced distance, this peak (2.24 ± 0.01 at 1.86 ± 0.01 Å across replicas) can be interpreted without shape-related ambiguity: its position coincides with the O···O separation of O–H···O hydrogen bonds and closely matches the hydrogen-bonded contact distances reported from MD simulations of glucose, maltose, and galactose/talose epimers [20,21,22], as well as from MDDF analyses of other polar solutes [31,32]. The first minimum occurs at approximately 3.51 Å, which we adopt as the boundary of the first hydration shell. In contrast, the conventional center-of-mass-referenced RDF (Figure 1a, red curve) shows only weak, broad variation over the same distance range, with no well-defined first peak. For an elongated tetrasaccharide, the center of mass does not coincide with any chemically meaningful site, so center-referenced RDFs smear the true contact distances into a nearly featureless curve; the MDDF, which requires no choice of reference center, avoids this ambiguity entirely.

Geometric counting via watershell analysis yields 69.5 ± 0.3 water molecules within 3.5 Å and 158.6 ± 0.7 within 5.5 Å (Table 1). Stachyose carries 14 free hydroxyl groups and seven additional ring and glycosidic oxygen acceptors, so the first shell corresponds to roughly five contact waters per hydroxyl group—or about three waters per polar oxygen—consistent with the multiple simultaneous contacts per polar group observed in disaccharide simulations [21,23] and with hydration numbers derived from ^17^O NMR and volumetric measurements on oligosaccharides [16,19]. The ratio of first-shell to total hydration-layer waters (~44%) indicates that the second shell (3.5–5.5 Å) remains significantly perturbed by the solute, in line with terahertz absorption experiments showing that mono- and disaccharides retard water dynamics over an extended range corresponding to several molecular layers [15,18]. Beyond 5.5 Å, water mobility approaches bulk-like behavior. Three independent observations support treating water beyond the second shell (r > 5.5 Å) as bulk-like. First, the MDDF decays to unity and shows no discernible structure beyond ~5.5 Å (Figure 1a), indicating the absence of any solute-induced density modulation at longer range. Second, the Kirkwood–Buff integral reaches a distance-independent plateau beyond 8 Å (Figure 1b), and recalculations with alternative bulk-range choices (12–16 Å and 15–20 Å) shift only the excluded-volume asymptote while leaving all short-range features unchanged. Third, residence-time analysis shows that even first-shell waters exchange with bulk on a ~42 ps timescale (Section 3.3), so any dynamical perturbation of the solute cannot propagate appreciably beyond the first two layers. This finite perturbation range is consistent with terahertz absorption experiments, which place the extended-range retardation of water dynamics by mono- and disaccharides at several molecular layers [15,18].

Notably, the KB integral evaluated at the bulk reference distance (8.0 Å, Figure 1b) is negative (−412 Å^3^). Within Kirkwood–Buff theory [43,44], a negative solute–solvent KB integral for a large neutral solute primarily reflects the excluded volume of the solute: the volume displaced by the tetrasaccharide dominates over the modest net accumulation of hydrating waters at long range, and a negative value is therefore expected rather than anomalous [32,45]. The physically meaningful hydration number is accordingly extracted from the short-range MDDF integral or from direct geometric counting, not from the asymptotic KB value, which converges to a volume-like quantity dominated by solute exclusion [31,44]. Calculations with alternative bulk-range choices (12–16 Å and 15–20 Å) shift the asymptote but leave all short-range features unchanged, confirming that the hydration-shell analysis is insensitive to the bulk reference.

### 3.2. Directional Hydrogen Bonding and Water Bridges

Hydrogen-bond analysis reveals an average of 21.1 ± 0.1 direct solute–water H-bonds per frame (Table 1). The directional asymmetry is modest but systematic: water acts as donor in 11.8 ± 0.1 bonds, while stachyose hydroxyls donate to water in 9.3 ± 0.1 bonds. This slight excess of water→solute donation arises from the four ring and three glycosidic oxygens, which can act only as acceptors and therefore recruit additional water donors beyond what the hydroxyl groups alone would require. A similar donor/acceptor imbalance was noted by Brady and Schmidt for maltose, where glycosidic and ring oxygens were found to compete with hydroxyl oxygens for water donation [21]. Averaged over the 14 free hydroxyl groups, the total count corresponds to about 1.5 hydrogen bonds per hydroxyl, which lies within the range of 1–2 reported for glucose, galactose, and talose [20,22] and for trehalose, maltose, and sucrose [23]. The higher absolute count simply reflects the larger number of polar sites on a tetrasaccharide (21 oxygen acceptors and 14 hydroxyl donors), and no directional preference emerges beyond that inherited from monosaccharide stereochemistry; GLYCAM06j/TIP3P thus reproduces the local hydrogen-bonding statistics essentially additively across the four residues.

**Figure 2:**
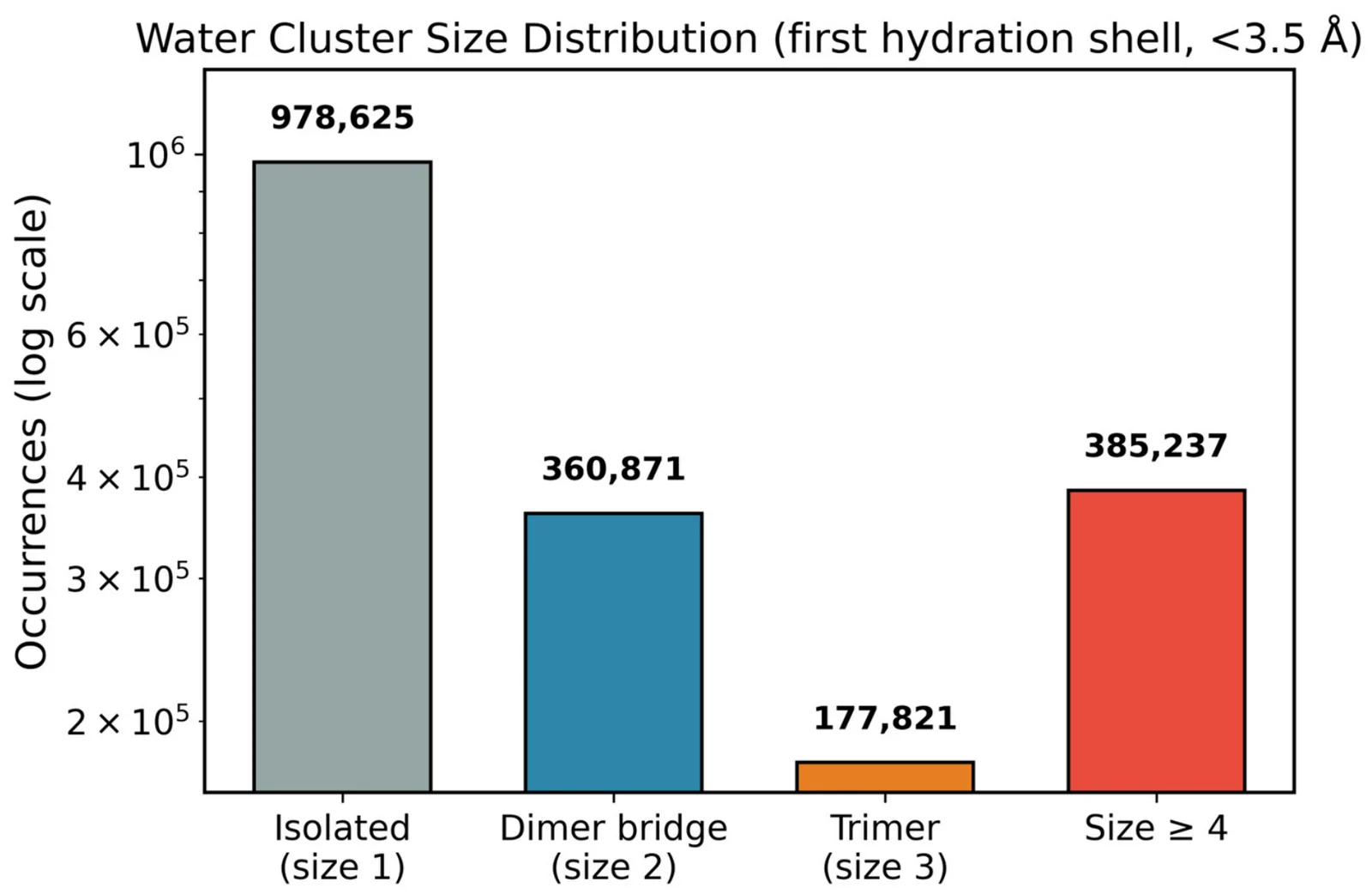
Water cluster analysis in the first hydration shell (<3.5 Å). Distribution of DBSCAN cluster sizes (ε = 3.0 Å, min_samples = 2) counted by occurrences across all frames of the three 300 ns replicas: isolated waters (51.4%), dimeric bridges (19.0%), trimers (9.3%), and clusters of four or more waters (20.3%). The largest instantaneous cluster spans 53–58 waters (replica-dependent), indicating that most of the first shell percolates into a single, rapidly exchanging hydrogen-bonded network. Counted by water molecules rather than by cluster occurrences, only ≈16% of the first-shell waters are isolated at any instant.

DBSCAN cluster analysis of the first-shell waters (<3.5 Å; ε = 3.0 Å, min_samples = 2) reveals a more nuanced picture (Figure 2). Counted by cluster occurrences across all frames of the three replicas, isolated waters account for 51.4% of observed clusters, dimeric bridges for 19.0%, trimers for 9.3%, and clusters of four or more waters for 20.3%. Within any single frame, however, these size classes coexist: on average only 10.9 ± 0.1 of the 69.5 first-shell waters are isolated, 4.02 ± 0.04 dimeric bridges span adjacent hydroxyl groups, and the remaining ~45 waters associate into a small number of larger aggregates. Throughout this work we designate the former population as “isolated” or “transient” first-shell waters: water molecules that reside within 3.5 Å of the solute but form no detectable water–water or water–solute hydrogen bond at our geometric criterion (3.0 Å, 135°). These molecules are anchored primarily by van der Waals and dipole–solute contacts rather than by directional hydrogen bonds, and they exchange with bulk on the tens-of-picoseconds timescale (Section 3.3). They should therefore not be regarded as a structurally distinct hydration species; rather, they constitute a continuously replenished pool from which the percolating hydrogen-bonded network is transiently assembled and into which it continuously dissolves.

Indeed, the largest instantaneous cluster comprises 53–58 waters depending on the replica—up to ~80% of the entire first hydration shell—showing that the shell is topologically percolated into a single hydrogen-bonded network rather than fragmented into independent, residue-local hydration patches. This connectivity must not be mistaken for structural order: residence-time analysis shows that individual waters exchange on a ~40 ps timescale (mean segment duration 41.8 ps; only 1.0% of segments exceed 200 ps), so the network remains compositionally fluid even where it is topologically connected. This picture contrasts with hydration in protein hydrophobic cavities, where geometric confinement forces water into ordered, long-lived clusters or chains with measurably slowed dynamics [46,47]. A recent VSFG-guided molecular model of protein hydration likewise identifies an intact, hydrogen-bonded first hydration shell as the hallmark of a stable protein surface [48]. The stachyose network reported here is percolated in the same topological sense—the largest instantaneous cluster spans ~80% of the first shell—so the contrast with protein hydration is one of timescale and surface chemistry rather than of connectivity. Size and surface morphology offer a natural explanation: on a small, uniformly hydrophilic, convex solute such as stachyose, the first shell is thin (~2 Å) and devoid of hydrophobic patches, and the densely distributed hydroxyl groups leave water–water association geometrically unconstrained and pervasive, so percolation is essentially guaranteed at all times—even as the network is continuously rewired by the large-amplitude jump mechanism through which liquid water reorients and exchanges hydrogen-bond partners on a picosecond timescale [49,50]; on protein surfaces, spanning hydration networks emerge only below a system-dependent temperature and are locally disrupted by exposed hydrophobic residues [51], so percolation there coexists with hydrophobicity-driven fragmentation.

The sensitivity of these shell-based observables to the numerical parameters of the analysis is summarized in Table S4 of the Supporting Information: a ±0.2 Å shift in the first-shell boundary changes the shell population by less than 10%, the percolation of the first-shell network is retained at the reference connectivity radius and above, and the fast-exchange residence picture is unchanged when the residence shell is defined at 3.5 Å instead of 5.5 Å. From a functional standpoint, the fluidity of this percolated network means that stachyose presents its hydroxyl groups to potential binding partners (α-galactosidases, transporters, mucosal lectins) in an essentially “pre-dried”, directly accessible state: the bridging waters that can confer specificity and binding enthalpy in lectin–carbohydrate complexes [9,10] exist here only as fleeting topological motifs.

### 3.3. Dynamic Hydration Core: Residence Times

**Figure 3:**
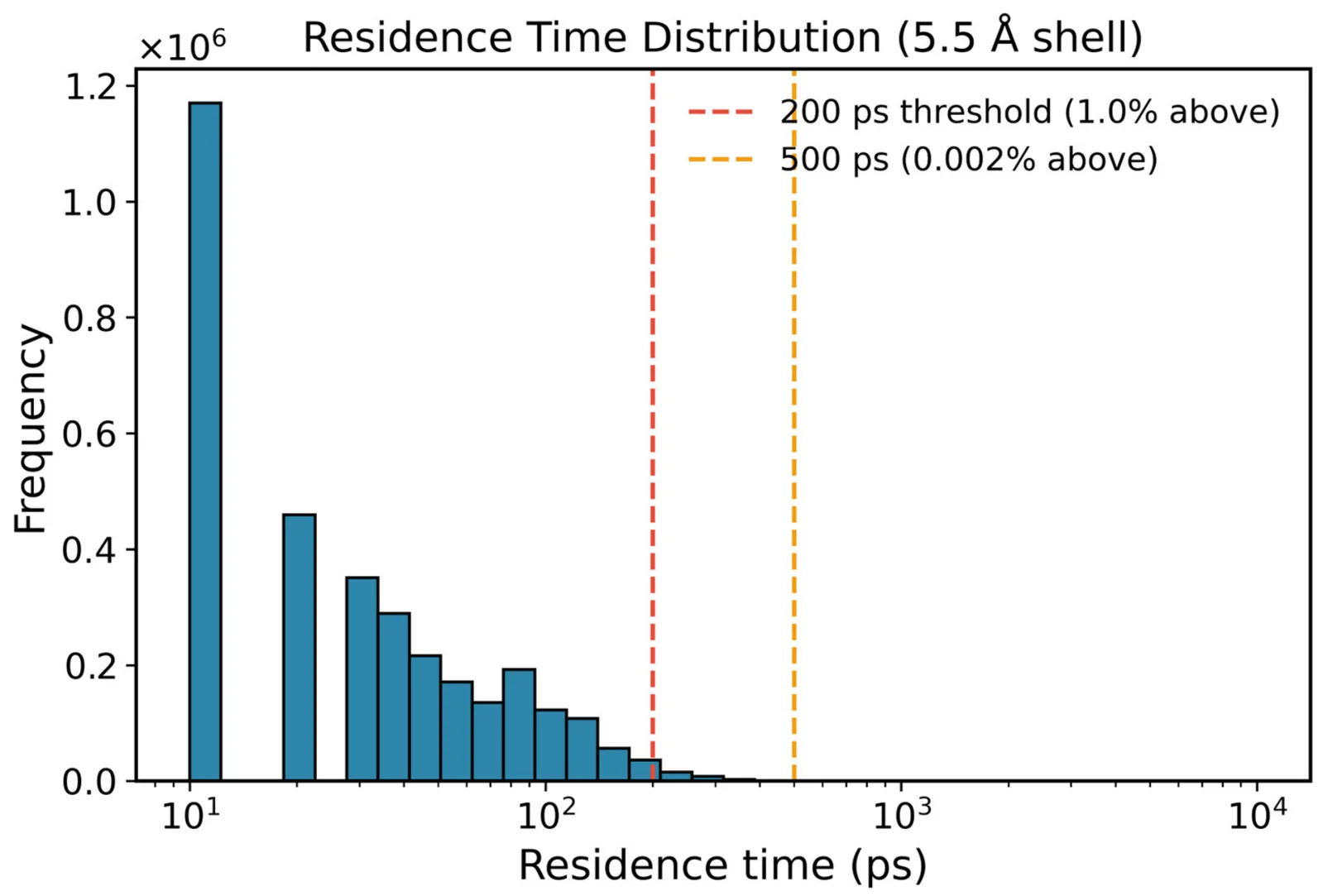
Residence-time distribution of hydration waters. Histogram of all continuous residence segments within the 5.5 Å shell on a logarithmic scale. The distribution is strongly skewed toward short durations (<100 ps), with only 1.0% of segments exceeding 200 ps and 0.002% exceeding 500 ps (mean segment duration 41.8 ps, median 30 ps). Segment boundaries tolerate gaps of ≤2 consecutive frames (Section 2.4).

While geometric counting identifies 158.6 waters in the 5.5 Å shell, only 11.1 ± 0.1 of these exhibit residence times exceeding 200 ps (Table 1). This “dynamic hydration core” constitutes roughly 7% of the geometric shell and ~16% of the first shell (3.5 Å). The residence-time distribution (Figure 3) is strongly skewed: the mean segment duration is ~42 ps, only 1.0% of all residence segments exceed 200 ps, and segments longer than 500 ps are exceedingly rare (<0.01%). These timescales bracket the values established for biomolecular hydration in general: residence times of interfacial waters range from a few picoseconds at exposed convex sites to several hundred picoseconds at concave, multiply hydrogen-bonded sites [39,40,50], and magnetic relaxation dispersion studies have shown that virtually all hydration waters exchange orders of magnitude faster than the solute reorients [28]. Stachyose, which lacks deep cavities entirely, sits at the fast-exchange end of this spectrum—as expected for a small, uniformly hydrophilic solute.

The disparity between geometric and dynamic hydration numbers carries important physical meaning. The majority of waters in the 5.5 Å shell are “transient visitors” with near-bulk exchange kinetics, whereas the ~11 long-residence waters constitute the structurally ordered layer that would dominate spectroscopic observables: terahertz absorption probes the ensemble of dynamically retarded waters [15,18], and NMR relaxation is weighted toward the longest-lived contacts [17,28]. This distinction resolves the apparent contradiction between MD-derived geometric hydration numbers (~70 for the first shell) and experimental reports of “hundreds of perturbed waters” per sugar molecule—the latter probes the large dynamical perturbation volume, which for disaccharides extends over several hydration layers [15], while the former counts tightly bound molecules. We also note that the TIP3P model is known to underestimate water viscosity and residence times somewhat [33,50], so our residence-time figures should be regarded as lower bounds; the qualitative conclusion—a small persistent core embedded in a fast-exchanging population—is robust to this bias.

### 3.4. Residue-Level Solvation Asymmetry

**Figure 4:**
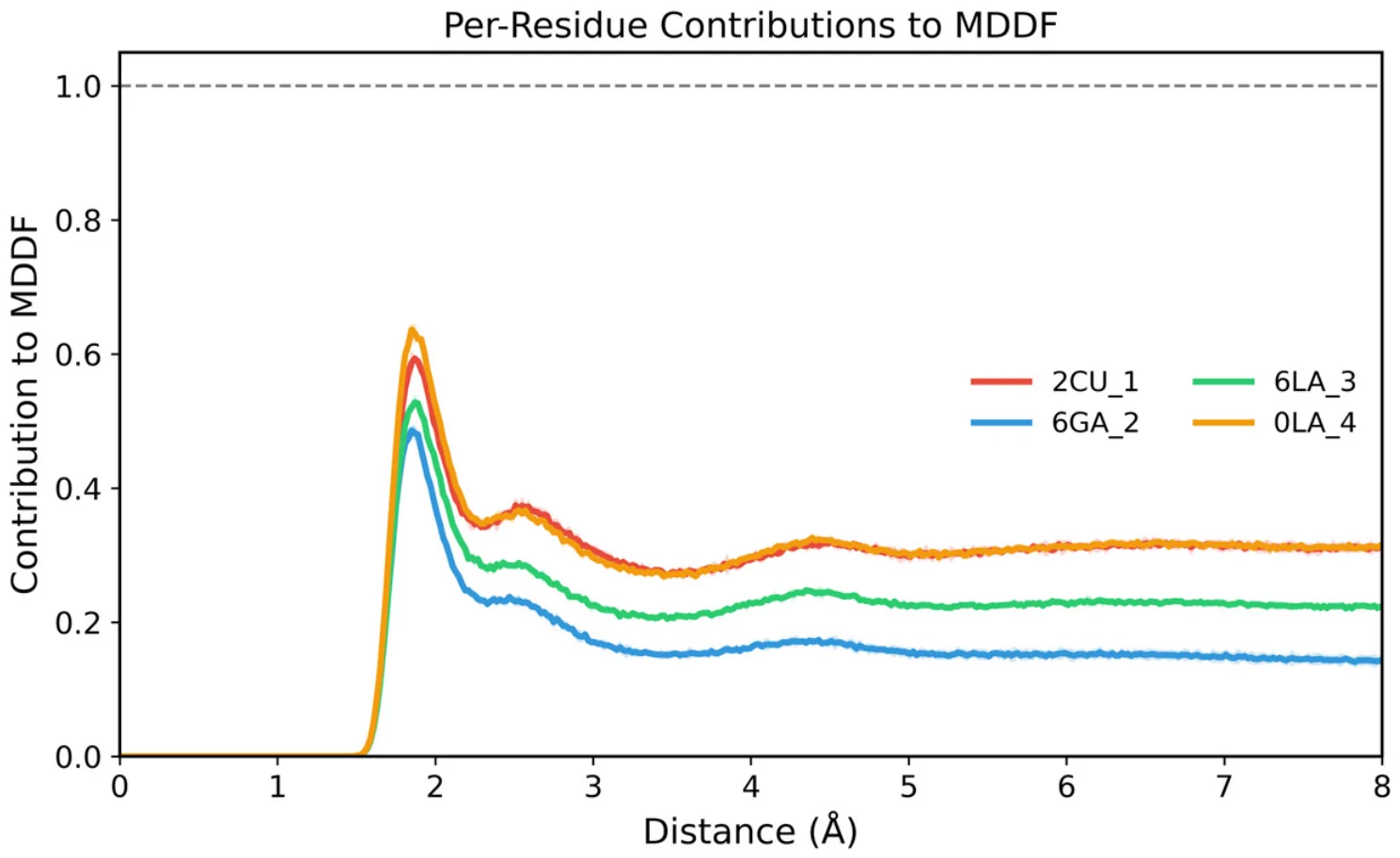
Residue-level hydration contributions to the MDDF. Decomposition of the first-shell MDDF into contributions from the four monosaccharide residues. Terminal residues (2CU_1, 0LA_4) account for ~58% of the first-shell integral. The horizontal dashed line at 1.0 denotes the bulk-density reference for the total MDDF.

**Figure 5:**
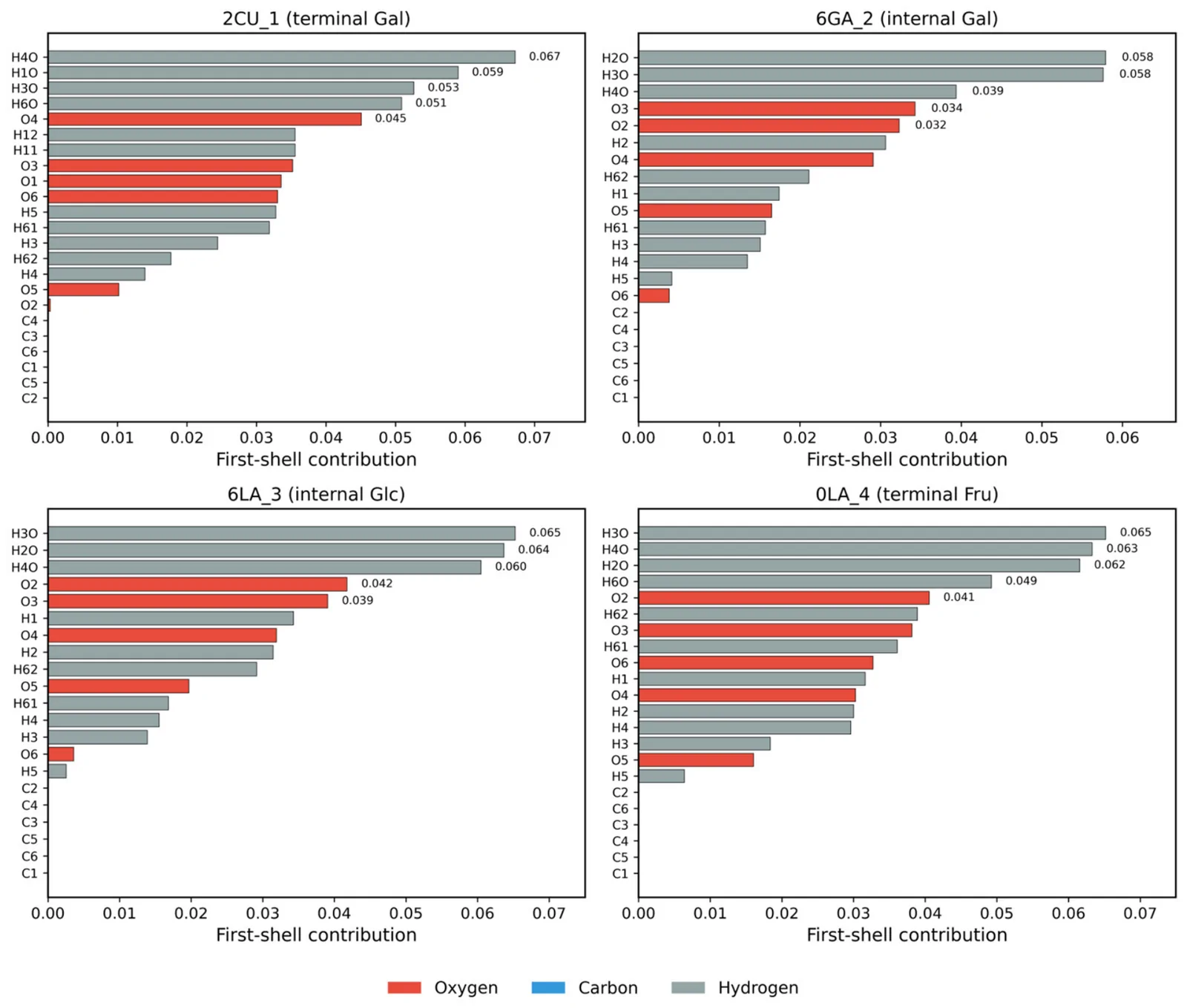
Atomistic hydration exposure map. Per-atom first-shell hydration contribution within each residue, ranked by magnitude. Oxygen atoms (red) and hydroxyl hydrogens (gray) dominate the high-exposure regions.

Decomposition of the MDDF into per-residue contributions reveals a marked asymmetry between terminal and internal monosaccharides (Figure 4). The terminal galactose (2CU, residue 1) and fructose (0LA, residue 4) together account for ~58% of the first-shell MDDF integral, despite representing only 50% of the solute atoms. Conversely, the internal galactose (6GA_2) and glucose (6LA_3) residues contribute 19.2% and 23.2%, respectively. This terminal-over-internal gradient is a direct consequence of the extended, antenna-like topology of (1→6)-linked oligosaccharides: internal residues are sterically shadowed by their neighbors along the chain, whereas terminal residues project freely into the solvent [7,8]. The per-atom decomposition (Figure 5) confirms that the high-exposure sites are predominantly hydroxyl oxygens and their hydrogens, with ring oxygens and C–H surfaces contributing little—consistent with the classical view that hydration of pyranose rings is dominated by the equatorial/axial arrangement of hydroxyls rather than by the ring skeleton [20,22].

**Figure 6:**
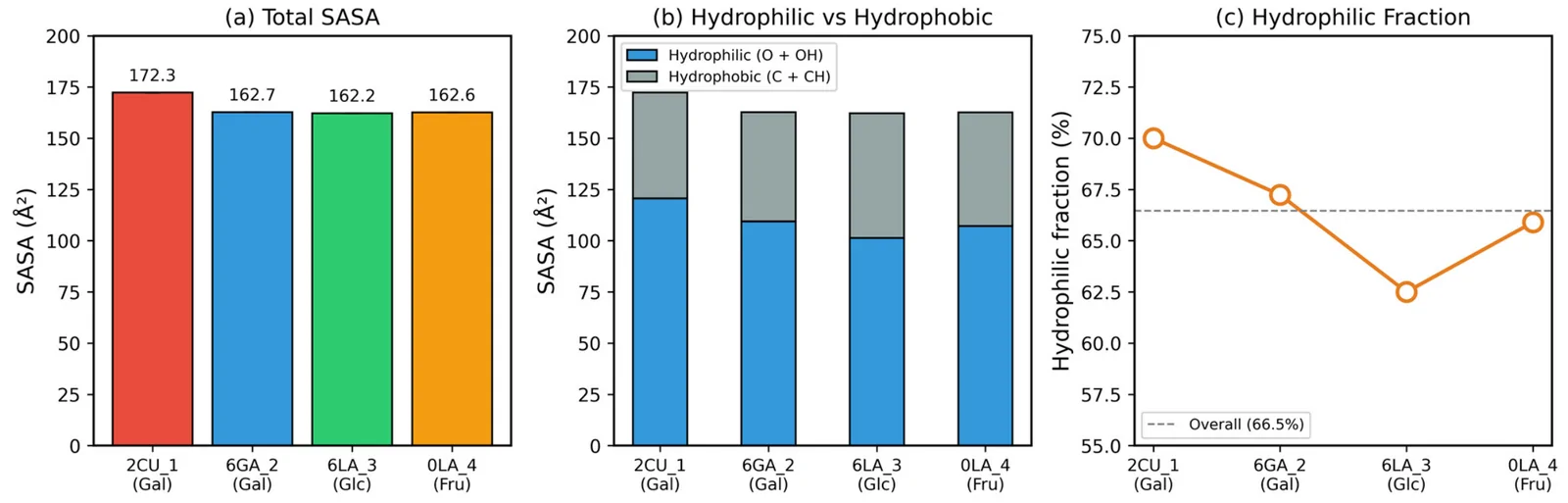
Solvent-accessible surface area decomposition. (**a**) Total SASA per residue, which is nearly uniform (162–172 Å^2^). (**b**) Stacked hydrophilic (O + OH, blue) and hydrophobic (C + CH, gray) contributions. (**c**) Hydrophilic fraction (%) per residue, ranging from 62.5% (6LA_3) to 70.0% (2CU_1).

SASA calculations show that static solvent exposure is remarkably uniform across the four residues (Table 2, Figure 6): per-residue SASA values span only 162.2–172.3 Å^2^, with the terminal galactose 2CU_1 (172.3 Å^2^) marginally the largest. The stacked hydrophilic/hydrophobic decomposition (Figure 6b) shows that all four residues are dominated by hydrophilic O/OH surface, with the hydrophobic C/CH contribution remaining below 40% in every residue. The hydrophilic fraction (Figure 6c) ranks the residues 2CU_1 (70.0%) > 6GA_2 (67.2%) > 0LA_4 (65.9%) > 6LA_3 (62.5%). Neither static descriptor, however, predicts the dynamic hydration ranking (0LA_4 ≈ 2CU_1 > 6LA_3 > 6GA_2): the fructose terminus 0LA_4 matches the galactose terminus in dynamic hydration (29.0% vs. 28.6%) despite a lower hydrophilic fraction (65.9% vs. 70.0%), and 6LA_3, the least hydrophilic residue, still surpasses 6GA_2 dynamically (23.2% vs. 19.2%). We attribute the anomalously high dynamic hydration of 0LA_4 to the conformational plasticity of the β-fructofuranose ring. Furanose rings are well known to pseudorotate freely among envelope and twist conformers on a low energy landscape [52,53], and pucker fluctuations transiently expose hydroxyl groups that are geometrically buried in the average structure, thereby enhancing dynamic solvent accessibility beyond what any static descriptor predicts. This finding carries a general methodological warning: static geometric descriptors such as SASA—however accurately computed [29,30,42]—are insufficient to rank carbohydrate hydration propensities, and dynamic sampling must be considered whenever flexible rings or linkages are present. It also rationalizes, at the residue level, why furanose-containing sugars behave differently from all-pyranose analogs in solution studies [23,52].

### 3.5. Conformational Sampling and Hydration Robustness

Conformational clustering of the combined 900 ns ensemble identifies four dominant families, together accounting for 89.2% of all frames. Cluster 0 (49.4%) corresponds to an extended conformation (heavy-atom radius of gyration ≈ 5.1 Å; end-to-end distance ≈ 10.7 Å) with both terminal residues projecting into solvent. Clusters 1 (17.8%) and 2 (11.8%) are bent states (Rg ≈ 4.5–4.8 Å; end-to-end distance ≈ 7 Å) arising from folding at the central α-1,6 linkages, and Cluster 3 (10.2%) is the most compact family (Rg ≈ 4.3 Å, both backbone bend angles > 100°) and also the most tightly defined (intra-cluster average distance 1.22 Å). The remaining six minor clusters (<5% each) are rare transient states. The prominence of extended conformations is consistent with the well-documented behavior of (1→6) linkages, whose additional ω torsion confers substantially greater conformational freedom than (1→2), (1→3), or (1→4) linkages and favors solvent-exposed, extended geometries in aqueous solution [7,8,11,22].

**Figure 7:**
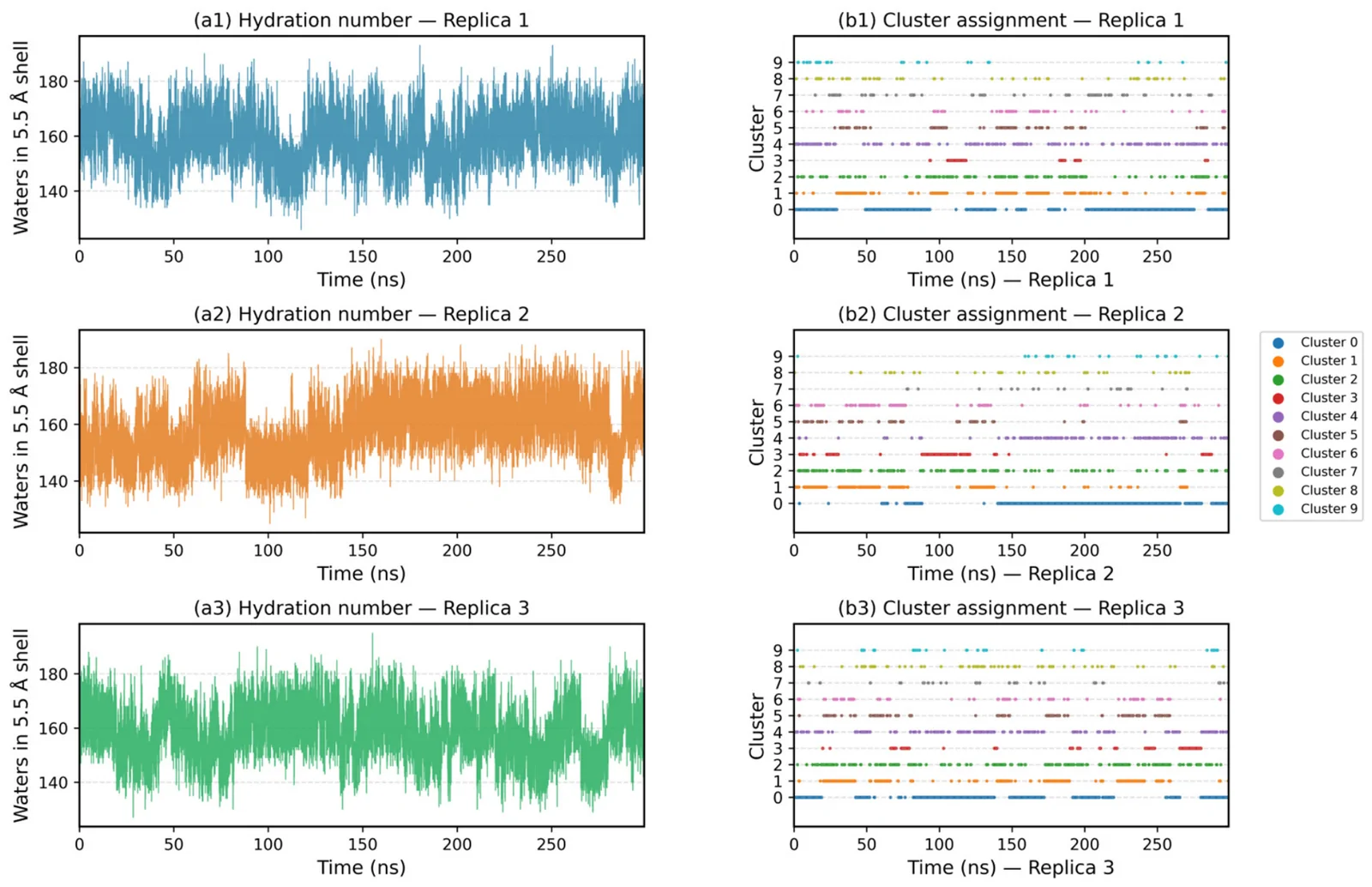
Conformation–hydration coupling for each independent replica. (**a1**–**a3**) Number of water molecules in the 5.5 Å geometric shell as a function of simulation time for Replicas 1–3, respectively. (**b1**–**b3**) Conformational cluster assignment versus time for each replica, displayed on its own time axis; cluster colors are consistent across all panels. The four dominant clusters (C0–C3) account for 89.2% of all frames, and the total hydration number remains relatively constant across sampled conformations in every replica.

The total geometric hydration number fluctuates around a mean of ~159 waters throughout each replica, with no dramatic correlation to global conformational transitions (Figure 7(a1–a3,b1–b3)). This insensitivity reflects the fact that stachyose’s surface remains largely solvent-exposed across all sampled conformations; even in the bent Cluster 1, the buried surface area is minimal. Transient dips in shell occupancy (to ~130 waters) occur across clusters and replicas alike, reflecting local exchange events rather than conformational gating. Two caveats deserve mention. First, conventional MD cannot exhaustively sample the glycosidic conformational space of a flexible tetrasaccharide. The present trajectories are not trapped in a single minimum: every replica visits all four dominant conformational families, the ω torsions of both (1→6) linkages interconvert between their rotamer states 76–165 times per replica (Figures S3–S5, Table S3), and a comparison with the classical MM3 steric-energy map of the 1→6 linkage [54] shows that the sampled (φ, ψ, ω) regions correspond to staggered minima of the map at all three ω rotamers, although the trans-ψ region favored in vacuum and in the crystal is not populated in aqueous solution (Figure S7). The sampling is therefore adequate for the local hydration observables targeted in this study—all of which are insensitive to the global conformational state (Figure 7)—but it cannot capture rare, slow rearrangements of multiple linkages, and enhanced-sampling or microsecond simulations might reveal more strongly coupled conformation–hydration states [27]. Second, carbohydrate force fields differ in their treatment of solute–solute interactions and partial charges, with known tendencies toward overstabilized aggregation or conformational collapse in some parameter sets [25,26]. The GLYCAM06j/TIP3P combination used here has been extensively validated for solution-phase carbohydrates [17,24,55], and our control simulations of sucrose (Section S1) confirm that this accuracy extends to the stereochemically demanding α(1→2)β linkage: the crystallographic conformation (108°, 305°) lies inside the global free-energy minimum, and the four characteristic basins of the reference GLYCAM06 landscape [36,56] are reproduced within 5°. A known bias of GLYCAM06—overstabilization of the second sucrose basin (M2), which our control simulations also reproduce (ΔF(M2−M1) ≈ 0.01 kcal mol^−1^)—does not affect any conclusion of this work, because all reported hydration observables are local, minimum-distance quantities that are insensitive to the relative populations of secondary glycosidic basins. The glucopyranose rings remain ^4^C_1_ throughout (Cremer–Pople θ = 9.8 ± 5.4°; 99.8% of frames below 30°), and the fructofuranose pseudorotation phase fluctuates around the crystallographic value (circular mean −11.2° versus −5° in the crystal), consistent with previous GLYCAM06 sucrose simulations [36]. Within the tetrasaccharide, the sucrose unit occupies a single, replica-reproducible φ–ψ basin that is shifted relative to isolated sucrose (Section S2); although we cannot exclude that conventional MD on the present timescale misses alternative (1→2) conformations, all reported hydration observables are local, minimum-distance quantities and are insensitive to which glycosidic basin is occupied (Figure 7). Neither caveat affects the central conclusion that hydration of stachyose is dominated by residue identity and terminal exposure rather than by global backbone conformation.

## 4. Conclusions

This study presents the first atomistically resolved hydration map of stachyose, a physiologically significant tetrasaccharide, using the minimum-distance distribution function framework. Our multi-pronged analysis yields the following key conclusions:

1. Stachyose possesses a dense but thin first hydration shell (69.5 ± 0.3 waters within 3.5 Å), dominated by direct O–H···O hydrogen bonding (21.1 ± 0.1 H-bonds per frame), consistent with hydrogen-bond stoichiometries established for mono- and disaccharides. Although first-shell waters percolate into a single hydrogen-bonded network, its rapid compositional turnover (mean residence ≈ 42 ps) and the absence of long-lived ordered clusters indicate a uniformly hydrophilic surface without hydrophobic pockets, in contrast to confined hydration sites in proteins.
2. A dynamic hydration core of 11.1 ± 0.1 long-residence waters (>200 ps) exists within the broader geometric shell. These waters likely mediate the spectroscopic and thermodynamic signatures observed in terahertz and NMR relaxation experiments, while the remaining shell waters behave as transient, bulk-like spectators.
3. Terminal residues exhibit higher solvent exposure than internal ones, but static SASA fails to capture the full picture: the fructose terminus (0LA) shows a dynamic hydration contribution matching that of the galactose terminus (29.0% vs. 28.6%) despite a lower hydrophilic fraction (65.9% vs. 70.0%), owing to furanose ring-flexibility-induced transient exposure.
4. The total geometric hydration number is robust to modest backbone fluctuations, suggesting that environmental perturbations of glycosidic linkage rotamers would alter stachyose’s solubility and enzymatic accessibility only weakly.

The computational workflow demonstrated here—combining MDDF decomposition, directional H-bond analysis, residence-time measurements, and conformational clustering—is readily transferable to other complex carbohydrates, polysaccharides, and glycoconjugates, where traditional RDF-based approaches fall short. Looking ahead, enhanced-sampling schemes such as replica exchange, together with a systematic comparative series across the raffinose family (raffinose, stachyose, verbascose) and deoxy analogs, would connect the local hydration picture established here to the global conformational thermodynamics of raffinose-family oligosaccharides.

## Figures and Tables

**Table 1:** Hydration parameters of stachyose at 300 K. Values are means ± standard deviation over three independent 300 ns replicas.

Parameter	Value	Method
MDDF peak height	2.24 ± 0.01	ComplexMixtures.jl
MDDF peak position	1.86 ± 0.01 Å	ComplexMixtures.jl
First-shell boundary	3.51 ± 0.05 Å	MDDF first minimum
Waters in 3.5 Å shell	69.5 ± 0.3	cpptraj watershell
Waters in 5.5 Å shell	158.6 ± 0.7	cpptraj watershell
Direct H-bonds (total)	21.1 ± 0.1	cpptraj hbond
Water→solute H-bonds	11.8 ± 0.1	cpptraj hbond
Solute→water H-bonds	9.3 ± 0.1	cpptraj hbond
Long-residence waters (>200 ps)	11.1 ± 0.1	MDAnalysis
Water bridges (Size-2 clusters)	4.02 ± 0.04 per frame	DBSCAN (first shell)

**Table 2:** Per-residue geometric and dynamic hydration properties. Dynamic hydration shares are normalized to the total first-shell MDDF integral and may not sum exactly to 100% owing to rounding.

Residue	SASA (Å^2^)	Hydrophilic Fraction (%)	Dynamic Hydration (%)	Key Feature
2CU_1	172.30	70.0	28.6	Terminal pyranose, dense hydroxyls
6GA_2	162.73	67.2	19.2	Internal, sterically shielded
6LA_3	162.19	62.5	23.2	Internal, partially exposed
0LA_4	162.64	65.9	29.0	Terminal furanose, conformationally plastic

## Data Availability

The AMBER topology (prmtop) and input files for all simulation stages, the ComplexMixtures.jl, cpptraj, and Python analysis scripts are provides as Supplementary Materials.

## Supplementary Materials

The following supporting information can be downloaded at: https://www.mdpi.com/article/10.3390/molecules31213799/s1, **Figure S1.** (a) Two-dimensional φ–ψ free-energy map of isolated sucrose from the combined GLYCAM06j/TIP3P control simulations (3 × 300 ns), with the crystallographic conformation (108°, 305°) and the M1 basin position of Xia & Case (105.1°, 304.9°) marked; contour spacing 1 kcal mol^−1^. (b) One-dimensional free-energy profiles F(φ) of the three independent replicas, demonstrating converged sampling. **Figure S2.** Ring conformations in the sucrose control simulations. (a) Distribution of the fructofuranose Altona–Sundaralingam pseudorotation phase in the three replicas; the crystallographic phase (−5°, i.e., 355°) and the experimentally preferred NE quadrant (0–90°) are marked. (b) Distribution of the glucopyranose Cremer–Pople θ coordinate in the three replicas (θ = 0° for ^4^C_1_, θ = 180° for ^1^C_4_). **Figure S3.** Time series of the glycosidic torsions φ1, ψ1, and ω1 of the α(1→6) linkage between residues 0LA and 6LA of stachyose, for the three independent 300 ns replicas. **Figure S4.** Time series of the glycosidic torsions φ2, ψ2, and ω2 of the α(1→6) linkage between residues 6LA and 6GA of stachyose, for the three independent 300 ns replicas. **Figure S5.** Time series of the glycosidic torsions φ3 and ψ3 of the α(1→2)β (sucrose) linkage within stachyose, for the three independent 300 ns replicas. **Figure S6.** Two-dimensional φ3–ψ3 free-energy map of the sucrose unit within stachyose (combined 900 ns ensemble), shown on the same scale as Figure S1a, with the crystallographic conformation of isolated sucrose (108°, 305°) marked. **Figure S7.** φ–ψ distributions of the two α(1→6) linkages of stachyose (combined 3 × 300 ns), partitioned by the ω rotamer of the same linkage (columns: gg, gt, tg; percentages give the combined ω-rotamer populations). Top row: Gal-α-(1→6)-Gal linkage (φ1, ψ1); bottom row: Gal-α-(1→6)-Glc linkage (φ2, ψ2). The star marks the stachyose pentahydrate crystal conformer as plotted on the MM3(92) isomaltose map of Dowd et al. (φ ≈ −45°, ψ ≈ 175° on the gg panel in the hydrogen-based convention of that work), converted to the present heavy-atom convention (φ ≈ 75°, ψ ≈ 175°). Torsion definitions are given in Section S2.1. **Table S1.** Positions and populations of the four φ–ψ basins of sucrose in the GLYCAM06j/TIP3P control simulations (combined 900 ns ensemble), compared with the reference GLYCAM06/TIP3P values of Xia & Case (Part 1). Populations are means ± standard deviation over the three replicas where applicable. The crystallographic conformation (108°, 305°) lies inside basin M1. **Table S2.** Ring-puckering statistics of the sucrose control simulations (89,700 frames). **Table S3.** ω-rotamer populations and numbers of rotamer transitions in the three stachyose replicas. Rotamers are assigned to the nearest of the gg (300°), gt (60°), and tg (180°) centres; transitions are counted between state segments with a minimum residence of 10 frames (100 ps) to exclude boundary recrossings. **Table S4.** Sensitivity of the key observables to the geometric parameters of the analysis (means over the three replicas). None of the conclusions of the main text is affected: the shell population varies by less than 10%; the absolute hydrogen-bond count depends on the distance criterion, as is well documented for geometric definitions, while all comparisons in the main text are made at a fixed criterion; the percolation of the first-shell network is retained at the reference connectivity (ε = 3.0 Å) and strengthened at ε = 3.2 Å, and moves toward fragmentation only under the stricter ε = 2.8 Å connectivity, consistent with the size/connectivity discussion in Section 3.2 of the main text; and the fast-exchange residence picture is unchanged at a 3.5 Å shell cutoff [57].
