# Supplementary material for: Atomistic Insights into the Hydration Structure of Stachyose: A Minimum-Distance Distribution Function Approach
Source: Molecules. 2026 Oct 22;31(21):3799. doi: 10.3390/molecules31213799 (PMC13765410; doi:10.3390/molecules31213799)
Supplement: Supplementary file 1 [file molecules-31-03799-s001.zip › molecules-4619349-supplementary.pdf]

Supporting Information

for

Atomistic Insights into the Hydration Structure of Stachyose: A Minimum-Distance Distribution Function Approach

Xiankai Jiang, Yuqiao Shen, Xiaolei Yin, Changying Wang, Junjian Miao*

Contents: S1. Control simulations of sucrose: validation of the α(1→2)β linkage (Figs. S1–S2, Tables S1–S2). S2. Glycosidic torsion sampling in stachyose (Figs. S3–S7, Table S3). S3. Sensitivity of the results to geometric parameters (Table S4). S4. Input files, topology, and analysis scripts.

S1. Control Simulations of Sucrose: Validation of the α(1→2)β Linkage

S1.1 Simulation protocol

An isolated sucrose molecule (α-D-Glcp-(1→2)-β-D-Fruf) was built with the GLYCAM06j force field and solvated in a cubic TIP3P water box with a minimum solute–box clearance of 15.0 Å. After energy minimization and NVT/NPT equilibration, three independent production replicas of 300 ns each (900 ns in total) were collected with settings identical to the stachyose production runs: NPT ensemble at 300 K and 1 bar, Langevin thermostat (γ = 2.0 ps^{-1}), Monte Carlo barostat, 2 fs time step, SHAKE constraints on all bonds involving hydrogen, 10.0 Å real-space cutoff with particle-mesh Ewald electrostatics. Coordinates were saved every 10 ps, and the first 1 ns of each replica was discarded, leaving 29,900 frames per replica for analysis.

S1.2 Torsion and puckering definitions

Glycosidic torsions are reported in the heavy-atom convention, identical to that of the reference GLYCAM06 sucrose study (Xia & Case, Part 1)[36]: φ = O5g–C1g–O2f–C2f and ψ = C1g–O2f–C2f–O5f, where the glycosidic oxygen is the fructose O2. In this convention the crystallographic conformation of sucrose is (φ, ψ) = (108°, 305°). Note that definitions based on hydrogen atoms (e.g., φH = H1g–C1g–O2f–C2f) differ from the heavy-atom convention by approximately ±120°, and that some earlier publications interchange the φ/ψ labels; all values reported here follow the heavy-atom convention above. The glucopyranose ring conformation was monitored through the Cremer–Pople puckering coordinate θ (θ = 0° for $^4C_1$, θ = 180° for $^1C_4$), and the fructofuranose ring through the Altona–Sundaralingam pseudorotation phase angle (0–360°), with the crystallographic phase at −5° (≡ 355°).

S1.3 The ϕ–ψ free-energy landscape reproduces the reference GLYCAM06 basins and the crystal conformation

The two-dimensional φ–ψ free-energy map computed from the combined 900 ns ensemble (Fig. S1a) shows four characteristic basins whose positions agree with the reference

GLYCAM06/TIP3P values of Xia and Case [36] within 5° (Table S1). Most importantly, the crystallographic conformation (108°, 305°) lies inside the global-minimum basin M1, centred at (102.5°, 300.0°). The M1 population is 0.406 ± 0.008 across the three replicas (individual values 0.413, 0.408, 0.397), and the one-dimensional free-energy profiles F(φ) of the three replicas are nearly indistinguishable (Fig. S1b), demonstrating converged sampling at the force-field level.

The second basin M2 (67.5°, 282.5°) is populated at 0.401 ± 0.002, i.e., nearly isoenergetic with M1 (ΔF(M2−M1) ≈ 0.01 kcal mol^{-1}). This is a stronger overstabilization of M2 than that reported by Xia and Case for their 100 ns GLYCAM06/TIP3P trajectories (ΔF ≈ 0.55 kcal mol^{-1}) and confirms, for our specific protocol, the known tendency of GLYCAM06 to overstabilize this secondary basin. As discussed in Section 3.5 of the main text, none of the hydration conclusions of this work depends on the relative populations of secondary glycosidic basins.

Table S1. Positions and populations of the four φ–ψ basins of sucrose in the GLYCAM06j/TIP3P control simulations (combined 900 ns ensemble), compared with the reference GLYCAM06/TIP3P values of Xia & Case (Part 1). Populations are means ± standard deviation over the three replicas where applicable. The crystallographic conformation (108°, 305°) lies inside basin M1.

**Basin**	**(φ, ψ), this work**	**(φ, ψ), GLYCAM06/TIP3P ref. (Xia & Case)**	**Population, this work**
M1 (global min.)	(102.5°, 300.0°)	(105.1°, 304.9°)	0.406 ± 0.008
M2	(67.5°, 282.5°)	(69.9°, 285.6°)	0.401 ± 0.002
M3	(82.5°, 192.5°)	(86.8°, 194.9°)	0.026
M4	(90.0°, 55.0°)	(89.9°, 51.0°)	0.024

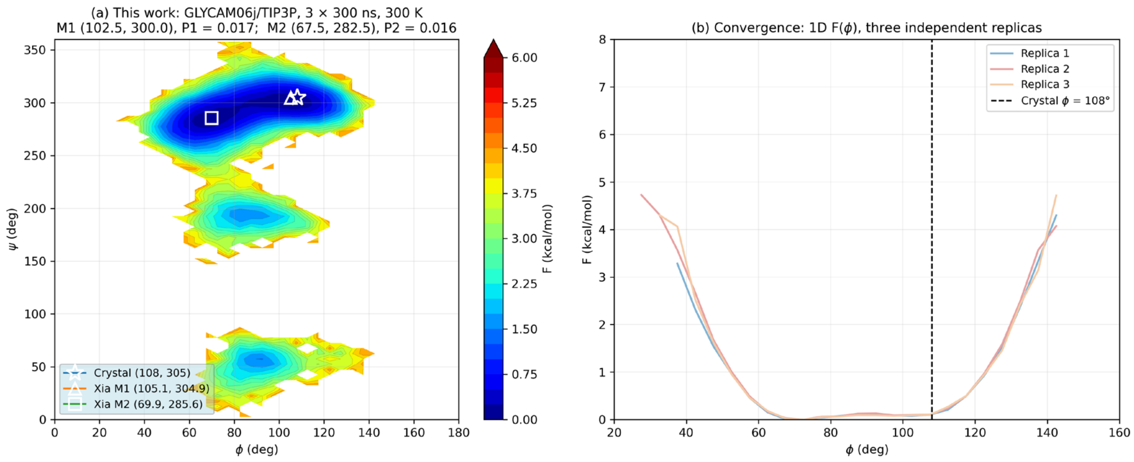

Figure S1. (a) Two-dimensional φ–ψ free-energy map of isolated sucrose from the combined GLYCAM06j/TIP3P control simulations (3 × 300 ns), with the crystallographic conformation (108°, 305°) and the M1 basin position of Xia & Case (105.1°, 304.9°) marked; contour spacing 1

kcal mol^{-1}. (b) One-dimensional free-energy profiles F(φ) of the three independent replicas, demonstrating converged sampling.

S1.4 Ring conformations: glucose remains $^4C_1$; fructose pseudorotates around the crystal phase

The glucopyranose ring remains in the $^4C_1$ conformation throughout all three replicas: the Cremer–Pople coordinate is θ = 9.8 ± 5.4° over the combined 89,700 frames, with 99.8% of frames below 30°. Rare transient excursions toward higher θ (at most 75.0°, in 0.01% of frames) are isolated single-frame events that immediately return to $^4C_1$ and never evolve toward $^1C_4$ (θ = 180°). The fructofuranose ring pseudorotates freely: the Altona–Sundaralingam phase has a circular mean of −11.2° (circular standard deviation 30.1°), with 64.5% of frames within ±20° of the crystallographic phase (−5°) and 28.9% of frames in the 0–90° (NE) quadrant; the mean puckering amplitude is 36.4°. This behaviour — fluctuation around the crystal phase with a bias toward negative phase angles relative to the experimentally preferred 0–90° quadrant — matches that reported by Xia and Case for GLYCAM06 sucrose simulations [36, 56].

Table S2. Ring-puckering statistics of the sucrose control simulations (89,700 frames).

Quantity	Value (combined 3 × 300 ns)
Glucose Cremer–Pople θ	9.8 ± 5.4°
Frames with θ > 30° / > 45°	0.19% / 0.01% (max 75.0°, transient)
Fructose pseudorotation phase, circular mean	−11.2° (circular SD 30.1°)
Frames within ±20° of crystal phase (−5°)	64.5%
Frames in 0–90° (NE) quadrant	28.9%
Mean fructose puckering amplitude	36.4°

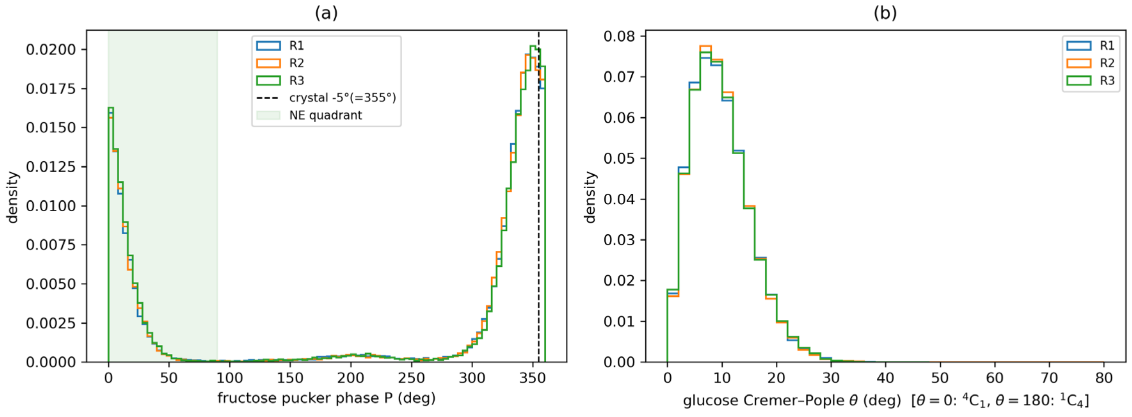

Figure S2. Ring conformations in the sucrose control simulations. (a) Distribution of the fructofuranose Altona–Sundaralingam pseudorotation phase in the three replicas; the crystallographic phase (−5°, i.e., 355°) and the experimentally preferred NE quadrant (0–90°) are

marked. (b) Distribution of the glucopyranose Cremer–Pople θ coordinate in the three replicas (θ = 0° for $^4C_1$, θ = 180° for $^1C_4$).

S2. Glycosidic Torsion Sampling in Stachyose

S2.1 Torsion definitions

Stachyose (0LA-6LA-6GA-2CU) contains two α(1→6) linkages and one α(1→2)β (sucrose) linkage. All torsions are reported in the heavy-atom convention:

Linkage 1→6 between residues 0LA and 6LA: φ1 = O5–C1–O6–C6, ψ1 = C1–O6–C6–C5, ω1 = O6–C6–C5–O5 (of residue 6LA). Linkage 1→6 between residues 6LA and 6GA: φ2, ψ2, ω2 defined analogously. Sucrose linkage between residues 6GA and 2CU: φ3 = O5g–C1g–O2f–C2f, ψ3 = C1g–O2f–C2f–O5f. The ω rotamer states are defined as gg (ω ≈ −60° / 300°), gt (ω ≈ +60°), and tg (ω ≈ 180°). Hydrogen-based definitions found in parts of the older literature [55] differ from these heavy-atom definitions by approximately ±120°, and some publications interchange the φ/ψ labels; the comparisons below are made only after conversion to the present convention.

S2.2 Time series of all glycosidic torsions

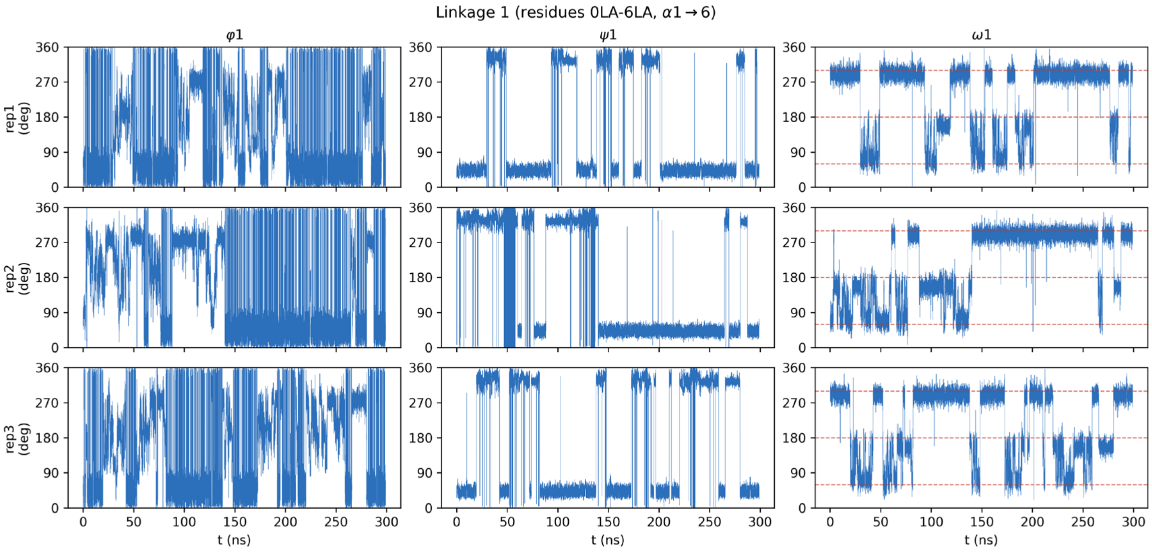

Figure S3. Time series of the glycosidic torsions φ1, ψ1, and ω1 of the α(1→6) linkage between residues 0LA and 6LA of stachyose, for the three independent 300 ns replicas.

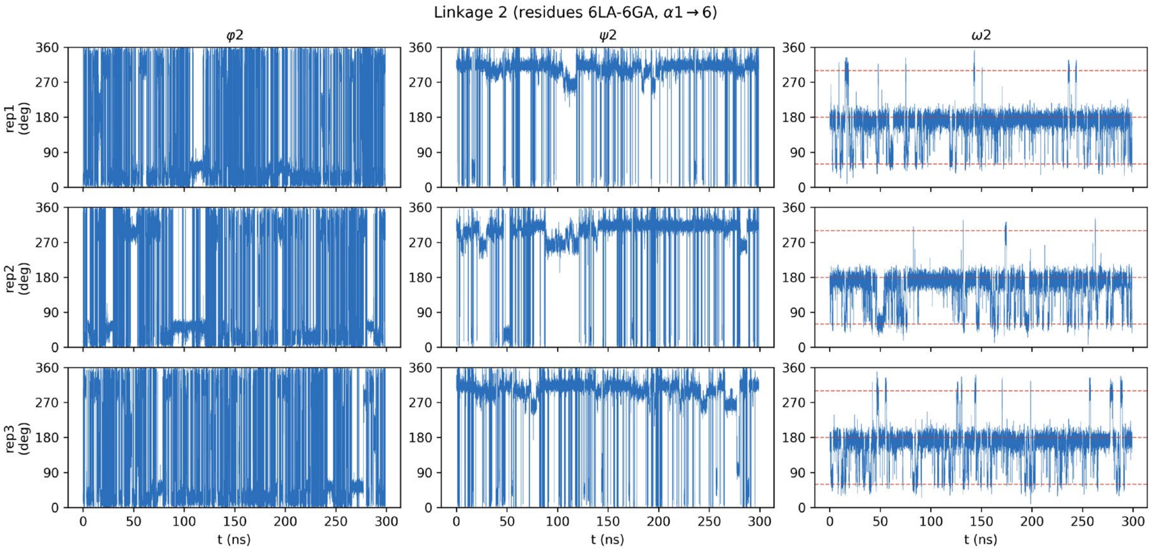

Figure S4. Time series of the glycosidic torsions φ2, ψ2, and ω2 of the α(1→6) linkage between residues 6LA and 6GA of stachyose, for the three independent 300 ns replicas.

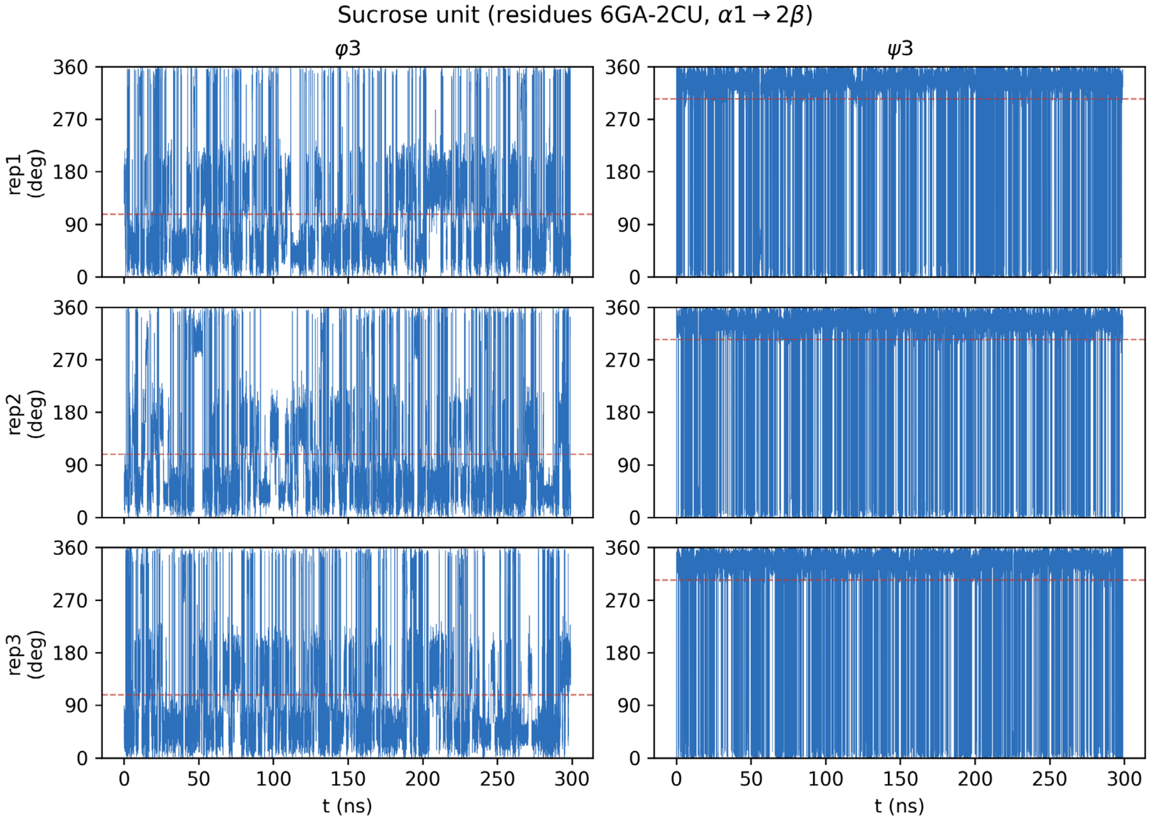

Figure S5. Time series of the glycosidic torsions φ3 and ψ3 of the α(1→2)β (sucrose) linkage within stachyose, for the three independent 300 ns replicas.

Within each 300 ns replica, the ω torsions of both (1→6) linkages interconvert frequently between their rotamer states: 76–96 transitions per replica for ω1 and 158–165 for ω2 (Table S3),

and every replica visits all four dominant conformational families (Fig. 7b of the main text). No replica remains confined to a single torsional minimum of either (1→6) linkage. The two linkages differ in their ω preferences: ω1 (on the galactose side of the first linkage) favours gg (58% combined), whereas ω2 (on the glucose ring carrying the (1→6) substituent) populates predominantly tg (80% combined).

Table S3. ω-rotamer populations and numbers of rotamer transitions in the three stachyose replicas. Rotamers are assigned to the nearest of the gg (300°), gt (60°), and tg (180°) centres; transitions are counted between state segments with a minimum residence of 10 frames (100 ps) to exclude boundary recrossings.

Replica	ω1 gg / gt / tg (%)	ω1 transitions	ω2 gg / gt / tg (%)	ω2 transitions
1	65.7 / 17.8 / 16.5	91	2.9 / 14.0 / 83.2	159
2	54.4 / 19.6 / 26.0	76	0.7 / 21.3 / 78.0	165
3	53.8 / 19.8 / 26.4	96	4.2 / 16.5 / 79.3	158
combined	58.0 / 19.1 / 23.0	—	2.6 / 17.2 / 80.2	—

S2.3 The sucrose unit within stachyose occupies a distinct, replica-reproducible basin

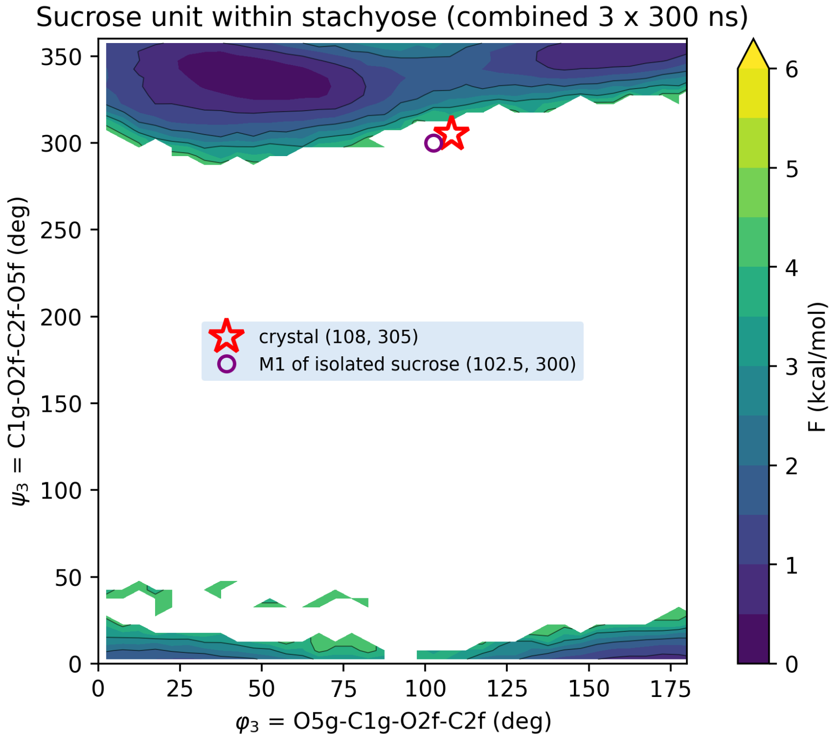

Figure S6. Two-dimensional φ3–ψ3 free-energy map of the sucrose unit within stachyose (combined 900 ns ensemble), shown on the same scale as Fig. S1a, with the crystallographic conformation of isolated sucrose (108°, 305°) marked.

Within stachyose, the sucrose unit does not populate the crystal-like M1 basin that dominates isolated sucrose (Section S1). Instead, all three replicas occupy a single, well-defined basin centred near (φ3, ψ3) ≈ (55°, 340°), which accounts for ≈59% of all frames, while the crystal-like M1 region is visited in only ≈1% of frames. The basin is a robust attractor: one replica leaves it during the first nanoseconds, explores other regions of the map, and then returns, after which all replicas remain for the full 300 ns. The preference of the (1→2) linkage is thus modified in the tetrasaccharide context, most plausibly through coupling to the (1→6) substituent on the same glucose ring, whose ω2 torsion populates the tg rotamer to ≈80% (Table S3). We note explicitly that 900 ns of conventional MD cannot exclude alternative (1→2) conformations; adaptive-biasing enhanced-sampling approaches [56] or the limitation stated in Section 3.5 of the main text apply here. All hydration observables reported in this work are local, minimum-distance quantities and are insensitive to which glycosidic basin is occupied (Fig. 7 of the main text).

S2.4 Comparison with the classical 1→6 steric-energy map

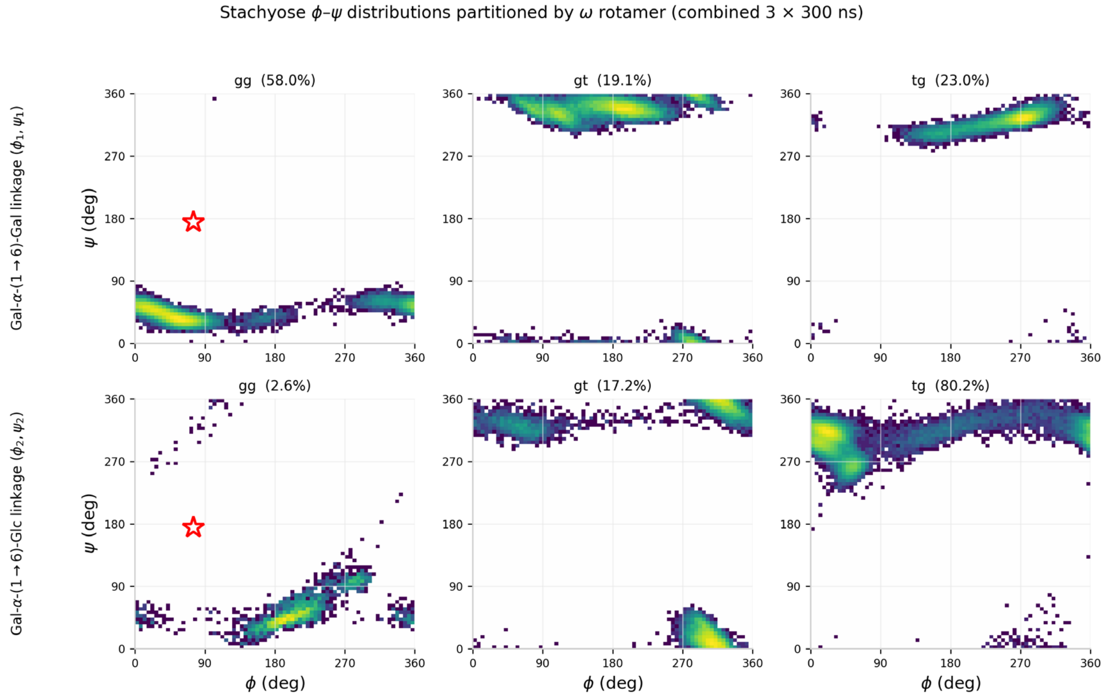

Figure S7. φ–ψ distributions of the two α(1→6) linkages of stachyose (combined 3 × 300 ns), partitioned by the ω rotamer of the same linkage (columns: gg, gt, tg; percentages give the combined ω-rotamer populations). Top row: Gal-α-(1→6)-Gal linkage (φ1, ψ1); bottom row: Gal-α-(1→6)-Glc linkage (φ2, ψ2). The star marks the stachyose pentahydrate crystal conformer as plotted on the MM3(92) isomaltose map of Dowd et al. (φ ≈ −45°, ψ ≈ 175° on the gg panel in the hydrogen-based convention of that work), converted to the present heavy-atom convention (φ ≈ 75°, ψ ≈ 175°). Torsion definitions are given in Section S2.1.

The MM3(92) adiabatic steric-energy map of isomaltose of Dowd, Reilly, and French [54] shows low-energy regions at all three staggered ω rotamers, with the three lowest minima at trans ψ

(≈180°) and higher-energy minima at the two gauche-ψ staggered orientations; the stachyose pentahydrate crystal conformer lies in the trans-ψ, gg region (star in Fig. S7). For the comparison, their hydrogen-based definitions (φ = H1–C1–O6–C6, ω = O6–C6–C5–H5) were converted to the present heavy-atom convention (φ_heavy = φ_H + 120°; ψ identical; ω_heavy = ω_H + 120°).

Our aqueous trajectories populate the gauche-ψ staggered regions at all three ω rotamers (Fig. S7), consistent with the gauche-ψ populations reported in GLYCAM06 Ramachandran-type analyses of α(1→6)-linked disaccharides [55] — corresponding to the secondary minima of the vacuum map — but do not visit the trans-ψ global basin (no frame within ±40° of ψ = 180°). Two factors account for this difference: (i) the reference map is a vacuum steric-energy map of an isolated disaccharide, whereas our simulations treat the full tetrasaccharide in explicit water; (ii) crystal conformers are subject to packing effects, as discussed by French and co-workers themselves [54]. Consistent with this, an NMR/MD study of stachyose in water reports linkage-specific α(1→6) solution conformations that differ between the Gal–Gal and Gal–Glc linkages [57]. We therefore use the vacuum map and the crystal conformer as references for the accessible regions of (φ, ψ, ω) space rather than for their solution populations; the residual limitation that conventional MD may miss rarely populated regions is stated in Section 3.5 of the main text.

S3. Sensitivity of the Results to Geometric Parameters

Key observables were recomputed at perturbed geometric settings to quantify their residual sensitivity to the numerical choices of the analysis.

Table S4. Sensitivity of the key observables to the geometric parameters of the analysis (means over the three replicas). None of the conclusions of the main text is affected: the shell population varies by less than 10%; the absolute hydrogen-bond count depends on the distance criterion, as is well documented for geometric definitions, while all comparisons in the main text are made at a fixed criterion; the percolation of the first-shell network is retained at the reference connectivity (ε = 3.0 Å) and strengthened at ε = 3.2 Å, and moves toward fragmentation only under the stricter ε = 2.8 Å connectivity, consistent with the size/connectivity discussion in Section 3.2 of the main text; and the fast-exchange residence picture is unchanged at a 3.5 Å shell cutoff.

Observable	Reference setting	Perturbed setting	Change
First-shell population	boundary 3.5 Å (MDDF first minimum 3.51 Å)	3.3 / 3.7 Å	−9.4% / +9.8% (63.0 / 76.3 vs. 69.5 waters)
Direct H-bonds per frame	3.0 Å / 135°	dist 2.8 / 3.2 Å	−49% / +22% (10.7 / 25.6 vs. 21.1)
Largest-cluster size	DBSCAN ε = 3.0 Å	ε = 2.8 / 3.2 Å	53–58 → 20–22 / 70–74 waters
Long-residence waters	shell 5.5 Å	shell 3.5 Å	1.0% → 0.18% of segments; mean

(> 200 ps)	segment 41.8 → 31.0 ps

S4. Input Files, Topology, and Analysis Scripts

The following files are provided as supplementary materials:

- AMBER topology (prmtop) and initial coordinate files for stachyose and sucrose;
- Input files for all simulation stages (minimization, NVT, NPT pre-equilibration, production) of both systems;
- ComplexMixtures.jl scripts for the MDDF and Kirkwood–Buff analyses;
- cpptraj input files for hydrogen-bond, solvent-shell, SASA, clustering, torsion, and puckering analyses;
- Python (MDAnalysis) scripts for residence-time and DBSCAN cluster analyses;
